# Stapled Hemorrhoidopexy: The Aga Khan University Hospital Experience

**DOI:** 10.4103/1319-3767.45358

**Published:** 2009-07

**Authors:** Ali Athar, Tabish Chawla, Pishori Turab

**Affiliations:** Department of Surgery, Aga Khan University, Stadium Road, Karachi, Pakistan; 1 Liaqat National Hospital & Medical College, Stadium Road, Karachi, Pakistan

**Keywords:** Hemorrhoids, stapled hemorrhoidopexy, high fiber diet, Milligan-Morgan open hemorrhoidectomy, Ferguson's closed hemorrhoidectomy

## Abstract

**Background/Aim:**

Stapled hemorrhoidopexy for prolapsing hemorrhoids is conceptually different from excision hemorrhoidectomy. It does not accompany the pain that usually occurs after resection of the sensitive anoderm. This study was carried out to evaluate the clinical outcome of stapled hemorrhoidopexy at The Aga Khan University Hospital.

**Materials and Methods:**

A sample of 140 patients with symptomatic second-, third-, and fourth-degree hemorrhoids and circumferential mucosal prolapse underwent stapled hemorrhoidopexy from July 2002 to July 2007. They were evaluated for postoperative morbidity, analgesic requirement, and recurrence.

**Results:**

Seventy-eight percent were males and the mean age was 45 (range 16-90) years. The mean operative time was 35 (15-78) min. The mean parenteral analgesic doses during the first 24 h were 2.1. All patients received oral analgesics alone after 24 h. No significant postoperative morbidity was observed. The mean in-patient hospital stay was 1.3 (0-5) days. Patients were followed-up for 24 (range, 2-48) months. Minor local recurrence of hemorrhoids was seen in four patients and was managed by band ligation.

**Conclusion:**

Stapled hemorrhoidopexy procedure was found safe, well tolerated by patients with minimal parenteral analgesic use and early discharge from the hospital.

Most symptomatic first- and second-degree hemorrhoids are successfully treated with a high-fiber diet supplemented with bulking agents such as Metamucil.[1] Surgical hemorrhoidopexy is reserved for prolapsing third- and fourth-degree hemorrhoids. It usually cures hemorrhoids, but excision of prolapsed hemorrhoids with conventional techniques (Milligan-Morgan[2] open hemorrhoidectomy, Ferguson's[3] closed hemorrhoidectomy) is a painful procedure. Patients experience postoperative pain because of the wide external wounds in the sensitive anoderm. Considerable postoperative nursing care is needed, with a convalescence of at least 1 month.

Stapled hemorrhoidopexy proposed by Antonio Longo in 1993[4] is a novel approach for the treatment of prolapsed hemorrhoids and external mucosal prolapse. This technique involves simultaneous excision and stapling of the circumferential column of the mucosa and submucosa in the insensitive area above the dentate line, resulting in reduction of mucosal prolapse. The excision interrupts blood flow from the branches of the superior hemorrhoidal artery, thereby reducing vascular congestion. These two are the suggested mechanisms by which stapling treats hemorrhoids. Excision does not involve hemorrhoidal cushions and so optimal continence is maintained. Several studies[5–8] have found that Longo's procedure is a simple, safe, and effective method that results in reduced postoperative pain, early recovery, and shorter hospital stay.

The present study evaluates the clinical outcome of stapled hemorrhoidopexy with regard to postoperative morbidity, analgesic requirement, and recurrence.

## MATERIALS AND METHODS

The study sample comprised of patients with symptomatic second-, third-, and fourth-degree hemorrhoids and circumferential mucosal prolapse, who underwent stapled hemorrhoidopexy in the Department of Surgery at the Aga Khan University Hospital, Karachi, from July 2002 to July 2007. Patients with thrombosed hemorrhoids, concomitant perianal fistula, fissures, or abscess and those undergoing a second procedure with same anesthesia were excluded from the study.

All patients were admitted on the day of operation without preoperative bowel preparation. Procedures were performed under general or epidural anesthesia and the operative position of patients was in lithotomy or prone Jackknife. Metronidazole (500 mg IV) and Cephalexin (1 gm IV) were given to all patients at the time of induction of anesthesia. Sigmoidoscopy was performed in selected patients before procedure to exclude any other pathology.

Stapled hemorrhoidopexy procedure was performed according to Longo's technique.[4] A circular anal dilator was placed and fixed. A purse string suture of 2/0 polypropylene was inserted at about 4 cm above the dentate line catching only the mucosa and the submucosa with the help of purse string suture anoscope. The distance of the purse string suture from the dentate line should be directly proportional to the extent of the prolapse so as to position the staple line at least 2 cm above the dentate line. A well-lubricated 33 mm stapling instrument (PPH 03, Ethicon Endo Surgery, Cincinnati, Ohio) with a fully opened position was then inserted and the anvil was positioned above the purse string. The purse string was snugged down on the shaft of the stapler and tied. The stapler was closed and fired and held closed for 30 s to aid homeostasis. In females, the posterior vaginal wall was checked before firing the stapler to prevent entrapment. The stapler was then fully opened to its maximum and gently withdrawn. The stapled line was inspected for bleeding and any spurting point was oversewn with Vicryl 3/0. Doughnut was checked and sent for histopathology.

Patient's demographic data, operative time, postoperative analgesic requirements, and complications during the hospital stay and during the follow-up visit were collected on a prospectively designed computer-based proforma. Data analysis was performed with the program Statistical Package for Social Sciences (SPSS Inc., Chicago, IL, USA). Descriptive statistics were computed and analyzed.

## RESULTS

A total of 140 patients were selected for the procedure of which, eight had symptomatic second degree, 118 had third degree, three had fourth degree and 11 had circumferential mucosal prolapse, respectively [Table 1]. Sixty percent had the disease for more than 1 year. Most of these patients had been treated by sclerotherapy and banding. The majority of the patients presented with bleeding and prolapse as their main complaints. Seventy-eight percent were males and 22% were females. The mean age was 45 years (range, 16-90). One hundred thirty patients (92%) were operated under general anesthesia and 10 (8%) under epidural anesthesia. The mean operative time was 35 min (range, 15-7). The mean duration of hospital stay was 1.4 days (range, 0-5). The mean duration of follow-up was 24 months (range, 2-48). Eight patients (5.7%) had postoperative bleeding, of which five patients had peroperative bleeding from the small bleeding points at the staple line, which were then oversewn. Three patients were readmitted with late postoperative bleeding.

**Table 1:** Degree of hemorrhoids

Hemorrhoids	No. of points	%
2^nd^ degree	8	5.7
3^rd^ degree	118	84.3
4^th^ degree	3	2
Circumferential mucosal prolapse	11	7.8

Postoperative analgesia during the first 24 h consisted of parenteral intramuscular injection (Diclofenic injection 75 mg or Pethidine 1 mg/kg) in all patients who underwent the stapled procedure. Seventy-seven percent of the patients received an injection of diclofenac and 33% received pethidine. The mean parental analgesic use during the first 24 h was 2.1 doses (range, 1-3). After 24 h, all patients were shifted to oral analgesics (Paracetamol 1 gm TDS) on an S.O.S basis.

Clinical outcome measures in terms of complications and recurrence are presented in Table 2.

**Table 2:** Postoperative complications

Complications	Patients
Bleeding	
Primary (minor/required transfusion)	5/1
Reactionary	0
Secondary	3
Retention of urine	2
Difficult evacuation	2
UTI	1
Anal fissure	4
Persistent pain and fecal urgency	4
Minimal residual prolapse	4
Technical problem	1

Discharge medication included were tablet Paracetamol 1 gm orally S.O.S and Isphagol husk at bedtime.

## DISCUSSION

Pain after hemorrhoidectomy has always been the main reason for patients to refuse surgery, whereas surgeons have a major concern for controlling postoperative pain. Multiple complementary treatments have been proposed to reduce postoperative pain, including the use of different surgical instruments (diathermy, scalpel, bipolar, scissors),[9] local[10] or systemic injection of analgesic,[11] antibiotics,[12] or associated procedures such as lateral internal sphincterotomy[13] to reduce postoperative sphincter spasm. All these treatments do not address the fact that the sensitive anal mucosa is severely traumatized during the removal of hemorrhoids. The operation proposed by Longo[4] does not damage the anal mucosa, which is lifted up in the anal canal by resection of a variable ring of insensitive mucosa above the anorectal junction.

The results of this study indicate that the postoperative morbidity was minimal. Molloy and Kingsmore[15] reported severe retroperitoneal sepsis after stapled anopexy and suggested routine antibiotic prophylaxis with this procedure. No patient in this study developed sepsis, except one who developed urinary tract infection as a result of postoperative urinary retention, which can be attributed to the catheterization. Prophylactic antibiotics in the form of Cephalexin and Metronidazole were given to all patients.

The incidence of early hemorrhage is not significantly lower than after conventional operation. Paolo Boccasanta *et al.*[5] have reported early and late bleeding in 12.5% of the patients who underwent stapled anopexy. We observed perioperative bleeding in eight (5.7%) patients and five of these patients had peroperative bleeding from the staple line, which was controlled with oversewing stitches. However, one patient required postoperative blood transfusion. The bleeding in this patient was the result of having a long-standing prolapse with a very thick and hypertrophied submucosa, which caused incomplete engagement of staples and partial disruption of the staple line. The incomplete staple line was oversewn. However, the blood loss had to be recovered by postoperative transfusion. One patient with chronic liver disease was readmitted after 1 week with late postoperative bleeding from the stapled line due to deranged coagulation. Bleeding was settled with correction of coagulation and blood transfusion without surgical intervention. Two other patients readmitted with bleeding were evaluated by proctoscopy but no active source was identified. Hence, both were treated conservatively.

Several randomized controlled trials[5–8] and a systematic review[16] have been published over the last 5 years. These trials have recruited 20-100 patients in each arm. The outcomes revealed in these studies were postoperative pain, the financial implications in terms of length of hospital stay and return to work, and functional outcome. Most of these studies have mentioned a learning curve of up to 10-25 procedures before the study. All, except the study by Shalaby *et al*.[17] have included third degree and mucosal prolapse. Our study includes 11% of the patients, a majority being females who had circumferential mucosal prolapse. We also included only those second-degree piles that failed an outpatient band ligation.

**Figure 1:**
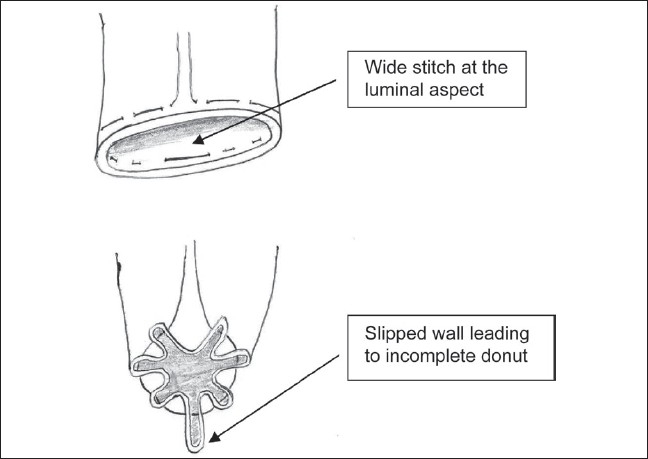
Wide stitch causes slipping of the rectal wall leading to incomplete donut

A systematic review and metaanalysis of the pooled data from seven randomized controlled trials with less than 1 year of follow-up comparing stapled hemorrhoidopexy with conventional hemorrhoidectomy showed that the total complication rates were similar for both techniques. It was noted that the stapled hemorrhoidopexy was associated with a higher rate of recurrent disease than conventional hemorrhoidectomy [RR 2.93 (95% CI: 1.41-6.07), P = 0·004; Heterogeneity I2 = 18.5%, P = 0·294]. Ganio *et al.*[19] have reported recurrent prolapse in 10 of 50 patients after stapled procedure. Ortiz *et al.*[20] reported it in seven of 27 patients in the fourth month of follow-up. In our series, localized residual prolapse was recognized at 6 months follow-up in four of 140 patients who had circumferential mucosal prolapse. This may be attributed to an incomplete doughnut in two patients. This occurs when the applied purse string suture skips a segment of the rectal wall [Figure 1] and hence the segment is not engaged in the stapling device. A useful technique[21] of applying purse string sutures without anoscope (fenestrated obturator) has been described recently. The technique ensures a circumferential grip on the rectal mucosa and avoids any skip area in the staple line. Another reason for an incomplete doughnut is due to the fact that the purse string had a slightly different entry and exit point, resulting in a spiral. This led to discontinuity in the doughnut even though it was a complete circle. This happened in the other two patients. Although the doughnut was a full circle, it was incomplete. Hence, minor localized recurrence seen in few initial patients with circumferential mucosal prolapse may be due to incomplete donut attributed to the technique of application of the purse string suture.

## CONCLUSION

In this study, the stapled hemorrhoidopexy procedure was found safe. It was well tolerated by patients with minimal parenteral analgesic use and early discharge from hospital. The morbidity observed was also low in this procedure. Based on our experience, we suggest use of stapled hemorrhoidopexy as a valid option for symptomatic second degree and all third- and fourth-degree hemorrhoids.
